# Evolution of the Concepts of Endometrosis, Post Breeding Endometritis, and Susceptibility of Mares

**DOI:** 10.3390/ani12060779

**Published:** 2022-03-19

**Authors:** Terttu Katila, Graça Ferreira-Dias

**Affiliations:** 1 Department of Production Animal Medicine, Faculty of Veterinary Medicine, University of Helsinki, 04920 Saarentaus, Finland; 2 Department of Morphology and Function, CIISA—Centre for Interdisciplinary Research in Animal Health, Faculty of Veterinary Medicine, University of Lisbon, 1300-477 Lisbon, Portugal; gmlfdias@fmv.ulisboa.pt; 3 Associate Laboratory for Animal and Veterinary Sciences (AL4AnimalS), 1300-477 Lisbon, Portugal

**Keywords:** mare, breeding, endometritis, endometrosis, inflammation, fibrosis, neutrophils, cytokines

## Abstract

**Simple Summary:**

Our understanding about inflammation of the endometrium after mating and susceptibility of mares to endometritis has changed in the last 100 years since it was recognized for the first time. Initially, it was believed that bacteria introduced into the uterus during mating could infect the uterus until it was shown that sperm induce neutrophilia. It was realized that post breeding endometritis (PBE) is a physiological defense mechanism used to clean the uterus from excess semen and inflammatory by-products. In mares susceptible to endometritis, PBE can be prolonged beyond the normal duration of 24 h. Delayed uterine clearance due to conformational defects, deficient myometrial contractions, and failure of the cervix to relax is detected by intrauterine fluid accumulation and is an important reason for susceptibility to endometritis. Untreated prolonged PBE can lead to bacterial or fungal endometritis called persistent or chronic endometritis. Multiparous aged mares are more likely to be susceptible. When sperm arrive in the uterus, pro-inflammatory cytokines are released. They attract neutrophils and induce modulatory cytokines which control inflammation. However, persistence of neutrophils and pro-fibrotic cytokines can have deleterious effects in inducing endometrosis. In this paper, the pathogenesis of fibrosis is reviewed. Endometritis and endometrosis are interconnected influencing each other.

**Abstract:**

In this paper, the evolution of our understanding about post breeding endometritis (PBE), the susceptibility of mares, and events leading to endometrosis are reviewed. When sperm arrive in the uterus, pro-inflammatory cytokines and chemokines are released. They attract neutrophils and induce modulatory cytokines which control inflammation. In susceptible mares, this physiological defense can be prolonged since the pattern of cytokine release differs from that of resistant mares being delayed and weaker for anti-inflammatory cytokines. Delayed uterine clearance due to conformational defects, deficient myometrial contractions, and failure of the cervix to relax is detected by intrauterine fluid accumulation and is an important reason for susceptibility to endometritis. Multiparous aged mares are more likely to be susceptible. Untreated prolonged PBE can lead to bacterial or fungal endometritis called persistent or chronic endometritis. Exuberant or prolonged neutrophilia and cytokine release can have deleterious and permanent effects in inducing endometrosis. Interactions of neutrophils, cytokines, and prostaglandins in the formation of collagen and extracellular matrix in the pathogenesis of fibrosis are discussed. Endometritis and endometrosis are interconnected, influencing each other. It is suggested that they represent epigenetic changes induced by age and hostile uterine environment.

## 1. Introduction

Our understanding about endometrosis, endometritis, and susceptibility to both has constantly evolved during the 100 years of endometritis research in mares. This review deals with the history of equine endometritis research and our current conception of post breeding endometritis. The pathophysiology of endometrosis and its connection with endometritis is discussed.

## 2. Susceptibility and Resistance to Endometritis

The conceptions of susceptibility and resistance to endometritis date to the year 1969, when Hughes and Loy discovered that young maiden mares cleared intrauterine bacterial inoculation rapidly (resistant) whereas aged multiparous mares remained chronically infected (susceptible) [1]. However, already in 1924, Dimock and Snyder had written that “infection of mares at the time of service is probably dependent upon some predisposition” [2].

In early endometritis studies, it was common to divide mares into resistant and susceptible categories based on their age, reproductive history, and Kenney biopsy category or histolopathological findings in biopsies [3,4,5,6,7]. Although these are important predisposing factors to endometritis, they do not always reflect susceptibility. Woodward et al. compared age, biopsy score, and fluid retention 48 h and 96 h after insemination with freeze-killed semen and reported that 36% of mares changed susceptibility status during subsequent seasons [8]. Only severe histopathological lesions in the endometrium were associated with susceptibility in the study of Troedsson et al. [9].

The next approach was the definition of the time required for uterine clearance after infusion of bacteria or different kinds of particles. Intrauterine bacterial inoculation of microspheres was cleared by the majority of resistant mares within 24 h, whereas susceptible mares failed to do so by 96 h [10]. Fumuso et al. used 72 h as the time point when resistant mares should be negative for intrauterine fluid (IUF), bacteriology and cytology after intrauterine inoculation of streptococci [11]. LeBlanc et al. infused charcoal into the estrous uterus, and if no charcoal was detected in uterine lavage fluid after 48 h, the mare was considered resistant [12].

The introduction of ultrasound examination enabled the detection of IUF accumulation [13], which is typical for susceptible mares after bacterial inoculation or mating [6,14,15]. Today, ultrasound examination of mares and appropriate treatment of susceptible mares after breeding is a routine procedure. Examination for the presence of IUF is the preferred method for practice and clinical field studies in diagnosing persistent post breeding endometritis. However, some mares without fluid can show polymorphonuclear leukocytes (PMN), and some others may show fluid without PMNs [16]. The detection of IUF >24 h after breeding suggests inadequate or delayed uterine clearance but not necessarily inflammation. Despite this, IUF is the most used and the most practical marker for susceptibility. Small amounts of fluid are normal during estrus but detection of ≥2 cm of IUF suggests susceptibility [15]. Approximately 14% of normal Thoroughbred mares in the USA have been reported to display moderate or large amounts of IUF after breeding [17]. In a similar study in UK this figure was 30% [14].

## 3. Post Breeding Endometritis

Dimock and Edwards (1928) cultured stallion semen and mare uteri after mating and concluded that mares were infected during breeding, streptococci being the most common bacteria [18]. After that, endometritis caused by mating, foaling, or conformational abnormalities was considered as the main cause of infertility of mares. In the 1980s, the main approach for research were intrauterine bacterial inoculations, the consequences of which were followed, and different parameters measured in mares which were divided into resistant and susceptible groups [5,6,9,12]. The bacterial challenge studies created a lot of useful basic knowledge about immunology and inflammation in bacterial endometritis.

In 1994, Kotilainen et al. infused different kinds of inseminates and semen extenders into the mare uterus instead of bacteria and reported that inflammation after breeding is induced by sperm [19]. Semen contains bacteria, but they are rapidly eliminated from the uterus by the intense but short-lived neutrophil influx [19,20]. Troedsson (1999) called this physiological phenomenon as post mating or post breeding endometritis (PME or PBE). Troedsson also used the term persistent post breeding endometritis (PPBE) to describe abnormally prolonged inflammation [21]. Normally PBE is over <24 h [20]; inflammation lasting >24–48 h is defined as PPBE. Thus, PBE is a physiological and short-lived defense mechanism, whereas PPBE is a prolonged reaction to mating. As the name implies, PBE and PPBE are inflammations, not infections [22,23], but PPBE can turn into bacterial or fungal endometritis [24] if not treated appropriately (lavage, oxytocin) [25,26]. However, the term persistent refers to chronic infectious endometritis, and therefore we suggest that PPBE should be called prolonged post breeding endometritis instead of persistent (Table 1). 

## 4. Delayed Uterine Clearance

Endometritis is a multifactorial disease. Uterine clearance equals to the mechanical pathway in the resolution of inflammation in addition to the innate immune response.

Delayed uterine clearance (DUC) of bacteria and inert, non-antigenic material was first reported in association with progesterone treatment and increasing age by Evans et al. in 1986 [27]. The authors suggested that physical clearance through the cervix plays an important role in the resistance of mares to uterine infection [28]. The existence of delayed uterine clearance in susceptible mares was confirmed by Troedsson and Liu (1991), who infused streptococci and non-antigenic microspheres into the uterus of susceptible and resistant mares [10]. Similarly, scintigraphy clearance studies by Le Blanc and her group demonstrated that susceptible mares had delayed expulsion of intrauterine radiocolloid as compared to resistant mares [12]. A tight cervix and inefficient uterine contractions are the major reasons for DUC.

### 4.1. Risk Factors

Conformational abnormalities are important risk factors to endometritis. Already in 1937, Caslick published his clinical findings on pneumovagina caused by the incompetence of the vulvar lips and vagino-vestibular sphincter associated with poor perineal conformation and underweight [29]. In 1990’s, LeBlanc and her group did many scintigraphy studies to prove the connection between conformational abnormalities and DUC in susceptibility [12]. In old multiparous mares, loss of the structural support of the caudal reproductive tract and stretching of the broad ligaments from repeated pregnancies may result in a uterus dropping and tilting ventrally in relation to the pelvic brim [30]. This may lead to urine pooling (accumulation of urine on the floor of the vagina) or pneumouterus [31]. Due to gravity, a pendulous uterus collects fluid that is difficult to evacuate [12,32]. Persistent IUF accumulations provide an appropriate environment for the growth of bacteria and can also be harmful to sperm [33]. Vascular elastosis may contribute to IUF accumulation through a reduction in endometrial perfusion and uterine drainage due to reduced venous return in capillary beds [26]. In addition, aged susceptible mares may suffer from inadequate lymphatic drainage that contributes to IUF. It has been suggested that lymph vessel dysfunction may be associated with endometrial fibrosis [34]. Endometrial fibrosis and biopsy scores IIB and III have been related to IUF [8,33,35] and susceptibility to bacterial endometritis [9]. Aging of the mare is an important risk factor for fibrosis, IUF, susceptibility, and fertility [3,4,36,37,38].

Manipulation of the cervix during insemination, presence of semen/fluid in the uterus, and prostaglandin F_2α_ (PGF_2α_) released from the endometrium and from cell membranes of activated PMNs induce uterine contractions after breeding [39,40,41]. It has been shown that susceptible mares differ from resistant mares in the electrical activity of the myometrium after bacterial challenge. The increase in activity occurred two hours later in susceptible mares compared to resistant mares and exhibited a sharp decline in activity at 12 h [41]. This is probably due to increased inducible nitric oxide synthase (iNOS), as nitric oxide relaxes smooth muscle. Susceptible mares have higher levels of intrauterine NO and increase in endometrial expression of iNOS mRNA [42,43].

After mating, uterine contents including excess semen and inflammatory by-products need to be evacuated rapidly [44]. Lymphatic drainage and myometrial contractions driven by PGF_2α_ play important roles. Myometrial contractions expel uterine contents through the cervix, and therefore, both an open cervix and strong myometrial contractions are important in uterine clearance [44,45]. Failure of the cervix to relax is encountered in both young and aged maiden mares and in some old multiparous mares [45,46]. Repeated foalings and manipulations of the cervix can result in cervical fibrosis and loss of elasticity with subsequent failure to dilate during estrus [45].

### 4.2. Intrauterine Fluid

All conditions listed above may occur in susceptible mares resulting in DUC. We see this as increased IUF after mating [14] or after bacterial challenge [6]. If the neutrophils and inflammatory by-products are not removed from the uterus, they will prolong the otherwise transient inflammation manifested as prolonged leukocytosis. Thus, DUC contributes to PPBE, but IUF is detected in estrus also before mating [33]. Estrogen increases endometrial secretion and edema during estrus. It has been shown that mares accumulating fluid during estrus have more glands with a larger diameter and wider lumens than mares without IUF [25]. This suggests that hypersecretion of glands could contribute to IUF accumulation. Tunòn et al. (2000) concluded that serum transudation is a major contributor for the formation of IUF diluting secretions of uterine glands [47]. Intrauterine fluid that we use as a diagnostic marker for PPBE originates from secretions of endometrial glands, from transudation and from failure of the mechanical uterine clearance, the latter being the most important one. In the studies of Pycock and Newcombe and Reilas et al., fluid collected from most mares had negative bacteriological and cytological results, and it was concluded that the fluid was not of inflammatory or infectious origin [14,33].

## 5. Physiology of Post Breeding Endometritis

When sperm arrive in the uterus, the local innate immune response is activated after antigen recognition. The antigens are presented to pattern recognition receptors (PRR) in the endometrial epithelial cells. Pathogen-associated molecular patterns (PAMP) are recognized by the Toll-like receptors (TLR) to initiate the inflammatory reaction [48]. Elweza et al. showed in cattle that spermatozoa bind to TLRs [48]. Presumably this also occurs in horses. In this in vitro study, sperm up-regulated dose-dependently interleukin 8 (IL 8), tumor necrosis factor α (TNFα), IL1β, nuclear factor kappa B2 (NFκB2), and complement factor 3 (C3) [49].

The innate immune response in endometritis has been described in detail in the excellent review of Canisso et al. [48]. Activation of TLRs initiates the inflammatory cascade which stimulates NFκB. The NFκB pathway activates genes coding for pro-inflammatory cytokines, chemokines, and cyclooxygenase-2 (COX-2). Pro-molecules of cytokines are activated by different molecules, but particularly by caspases [48]. In the equine endometrium, COX-2 is expressed after infusion of seminal plasma or semen extender [50]; furthermore, COX-2 induces a local endometrial increase in PGF_2α_, 16 h after breeding [51]. The major functions of the innate immune system are to recruit immune cells through activation of cytokines including chemokines and to activate the complement cascade to enhance the phagocytosis of damaged cells and microbes. Equine spermatozoa induce the complement cascade leading to an increase of C3b and C5a, leukotrienes, and prostaglandins (PG) resulting in the chemotaxis of PMNs into the uterus [48]. 

Pro-inflammatory cytokines activate vascular endothelial cells (arteriole constriction and venule dilation) increasing vascular permeability [48]. Transudate leaks to the interstitium causing edema and fluid accumulations. Alterations in the permeability lead to cellular responses. Chemotaxis of neutrophils is induced via P- and L-selectin. Neutrophils produce integrins, which bind to adhesion molecules on endothelial cells, and adhere to the blood vessel walls [48]. Neutrophils are detected in the uterine lumen within 30 min following artificial insemination (AI) and peak between 6 and 12 h [20,24]. The most important task of neutrophils is phagocytosis of sperm and bacteria, but they also secrete additional cytokines and chemotactic mediators, further contributing to inflammation, and release PGs important for myometrial contractility [48]. 

Neutrophils, as the first line of immune defense mechanism, are able to form neutrophil extracellular traps (NETs); this is a phagocytosis-independent mechanism. NETs are DNA strands surrounded by various cytoplasmic and nuclear proteins that trap and/or kill bacteria, spermatozoa, and parasites [52,53]. The activity of NETs, called NETosis, is due to the rupture of neutrophils and release of granules, allowing their chromatin to meet antigens and other immune cells. Proteins and enzymes found in NETS [52] serve as an additional antimicrobial mechanism, but they can also stimulate fibrosis establishment [51,54].

Innate immune response is nonspecific and acts as the first line of defense against pathogens, foreign stimuli that include constituents of seminal fluid, and local infections (endometritis). It has been recently established that in restraining bacteria, NETs formation is also involved in the pathogenesis of mare endometrial fibrosis (endometrosis). Moreover, persistent resident macrophages and mast cell activation could also have pro-fibrotic roles by secreting great amounts of pro-fibrotic factors and lead to fibrosis [55].

### Cytokines

Inflammatory mechanisms of endometritis have been reviewed by Woodward and Troedsson [56]. Potent pro-inflammatory cytokines IL1α and IL1β are released at the onset of inflammation and upregulate other pro-inflammatory cytokines. Interferon gamma (IFNγ) aids inflammatory cells to migrate through vessel walls and upregulates iNOS [56]. Normal mares showed high mRNA expression for IL1β, IFNγ, and chemokine IL8 at 2 and 6 h after AI [56,57]. Expression of TNFα was the highest at 2 h and that of IL6 at 6 h. Although IL6 is initially proinflammatory, it has also protective roles through the modulation of other pro-inflammatory cytokines and induction of modulating cytokines, such as interleukin-1 receptor antagonist (IL1RN). Pro-inflammatory cytokines can lead to exacerbated inflammation and tissue damage; therefore, they must be controlled by anti-inflammatory cytokines. In resistant mares, IL10 and IL1RN peaked at 6 h [56,57]. Susceptible mares presented lower expression of modulatory cytokines IL6, IL10 and IL1RN at 6 h than resistant mares. They had also higher neutrophil counts at all time points compared to resistant mares, but at 2 and 6 h the differences were significant. The data suggest that around 6 h after AI may be a critical time in developing susceptibility. A failure to resolve PBE in a timely fashion may be due in part to a failure to mobilize cytokines during the early Inflammatory period, which could contribute to a delayed resolution of inflammation in susceptible mares [56,57]. In the study of Fumuso et al. (2007), susceptible mares had significantly higher mRNA transcription of IL8 and significantly lower of IL10 at 24 h after AI as compared to resistant mares [11]. The inflammatory condition persisted in susceptible mares after AI until day seven post-ovulation [11]. Both studies show a delayed pattern of cytokine expression for susceptible mares, particularly for modulatory cytokines [11,57].

## 6. Treatments

The thorough review of Morris et al. presents the treatments of endometritis [24]. Ecbolic agents and uterine lavage target the mechanical pathway by aiding physical clearance. These valuable tools became common practice in the treatment of endometritis first in the 90’s. Allen introduced the use of oxytocin in the evacuation of uterine contents [58]. Uterine lavage got increasingly common with the embryo flushing techniques and turned out to be effective in reducing growth of bacteria and number of neutrophils [59]. According to Morris et al., “lavage in susceptible mares is indicated when there is hyperechoic intrauterine fluid accumulation or if free intrauterine fluid exceeds two cm in diameter” [24]. However, routine uterine lavages of all mares after mating are sometimes practiced. One should bear in mind that post breeding inflammation is a defense mechanism itself, normal mares have healthy mucus and mucociliary currents between endometrial folds to drain fluid, and mating is a natural event with which mares have been able to cope for thousands of years [60]. Unnecessary routine treatments should be abandoned, and treatments focused on mares that need it.

Immune modulators are our newest drugs: corticosteroids, platelet rich plasma, stem cells, Mycobacterium wall extracts [24]. Treatments of susceptible mares with prednisolone [61] or dexamethasone [62] after mating resulted in the decrease of IUF and increase in pregnancy rates. After intrauterine infusion of *Escherichia coli*, expression of pro-inflammatory cytokines (IL1β, IL6, IL8) was significantly lower in the mares treated with dexamethasone than in the non-treated group [63]. In addition, susceptible mares inseminated with killed sperm and treated with dexamethasone had significantly lower expression of IL1β at 6 h after AI, as compared to the non-treated cycle, but IL6, IFNγ, IL6, IL10, and IL1RA were not affected [64]. These studies indicate that deviations in cytokine expression after mating determine susceptibility. Furthermore, the effects of corticosteroid treatments on cytokine expression—decrease in pro-inflammatory and increase in anti-inflammatory cytokines—improve the cytokine imbalance in susceptible mares.

In Thoroughbred stud farms, a routine use of antibiotics to every mare after mating was practiced for a long time since it was believed that bacteria cause post breeding endometritis [14]. However, PBE is not associated with bacteria either in normal mares [19,20,65] or in mares with pathological endometrial changes [66]. Even the uterus of mares with DUC caused by the blocked cervix contains no bacteria [23]. Although there are bacteria in the semen, the quick and intensive PMN influx takes care of them within 4 to 12 h [20]. These studies confirm that administration of antibiotics is not indicated in the treatment of PPBE, at least not in the early stage. Exceptions are mares with a history of infectious endometritis or clear signs of it after AI. On the other hand, our sampling methods are not very sensitive, particularly the swabs [22]. Thus, we may fail to diagnose some asymptomatic mares with chronic endometritis or dormant streptococci [26,45]. However, the number of these mares is low, and does not justify routine antibiotic treatments. Each case must be clinically evaluated for the need of antibiotic administration considering the previous history of the mare.

## 7. Etiology of Prolonged Post Breeding Endometritis

When a mare faces a trauma during parturition, the vulvar lips, vulvovaginal sphincter, vagina, cervix, or uterus may suffer tears which can result in permanent conformational defects [31]. Multiple pregnancies stretch ligaments and the uterus, which may lead to a pendulous uterus tilting ventrally and further to fluid accumulations and delayed uterine clearance [30,32]. Due to repeated parturitions and manipulations, the cervix may become fibrotic and fail to dilate adequately during estrus [45]. This has been reported also in embryo donor mares which are exposed to frequent uterine flushes through a relatively large catheter that stretches the cervix [31,45].

### Occlusion of the Cervix

An experiment by Reilas et al. (2016) showed how mares can become susceptible after an insult to the uterus [23]. The experiment included three mare groups: (1) controls that were only inseminated, (2) mares whose cervix was occluded with a clamped catheter for 25 h after AI, and (3) mares having the catheter occluding the cervix opened and drained 6 h after AI and then closed again until 25 h. The mares underwent five cycles: in the 1st, 3rd, and 5th PG-induced cycle, swabs were taken to ascertain that the mares were free of bacteria and PMNs. In addition, endometrial biopsies were obtained during the first and last cycle. In the 2nd and 4th cycle, the mares were inseminated. The mares that had been treated during the 2nd cycle served as controls during the 4th cycle and vice versa. Two kinds of fluids were collected and analyzed: native undiluted fluids (catheter fluids (6 and 25 h) and tampon fluid (25 h) from the controls) and lavage fluids at 25 h [23].

As expected, the treatment groups accumulated significantly more fluid and PMNs than the controls in the first treatment cycle. Unexpectedly, during the 2nd treatment cycle, the controls did not differ from the treated groups since they had as much fluid, PMNs/mL and total PMNs (fluid volume × PMNs/mL) as the mares with the occluded cervix. Despite the presence of large amounts of IUF, none of the mares had bacterial growth. The 25-h fluids contained a lot of cytokines (IL1β, IL6, IL10, TNFα), but there were differences neither between the cycles nor between the groups. Periglandular fibrosis increased significantly during the experiment. The pregnancy rate was 2.5 times lower in the 4th estrus than in the 2nd estrus (17% vs. 42%) [23]. 

The occlusion of the cervix for 25 h after AI prevented all normal drainage of excess sperm, PMNs and inflammatory by-products. Their continued presence provoked more PMNs and cytokines denoting that the endometrium was exposed to inflammatory media for 25 h. The large volume lavage was the only treatment, but the mares were negative for PMNs and bacteria in cytology in the next estrus induced by PG on Day 15 [23]. 

The catheter fluids represent accumulation of cytokines during different time frames, and therefore, it is impossible to determine, if there was a delay or deficiency or abundance of certain cytokines. Cytokine expression of susceptible mares differs from that of resistant mares, being somewhat delayed, particularly for anti-inflammatory cytokines, which suggests that the inflammation in susceptible mares can be more intense and/or prolonged; this is also shown by PMN numbers [57]. It is probable that the cytokine release in the cervix occlusion study changed its pattern from the first treatment cycle to the second treatment cycle. It is speculated that changes in gene expression could be epigenetic changes induced by the hostile/highly inflammatory uterine environment. It is not known if this change persists.

The previous literature implicates that the inflammation must be over by the time the embryo arrives in the uterus, five to six days post ovulation, to achieve normal pregnancy rates [45]. However, it seems that already inflammation extending over 24 h is harmful for the endometrium resulting in long acting or even permanent changes, such as fibrosis, and low pregnancy rates. This emphasizes the need for timed examinations (6 to 12 h after breeding) and a quick treatment to aid uterine clearance in problem mares [44].

## 8. Endometrosis

In his pioneering publication in 1978, Kenney divided chronic histological changes in equine endometrial biopsies into inflammatory and degenerative [3]. Later those chronic degenerative changes in the endometrium, which are responsible for infertility mainly in older mares, were started to be called endometrosis [67,68].

Even though mare endometrial biopsy is considered as safe and practical [3] and used as a routine standard procedure in the breeding examination of problem mares and in research experiments, there are some pitfalls. The histopathological grading system of Kenney and Doig, based on several criteria, such as inflammation, gland density, dilation, and nesting, and fibrosis, among others, is in general use [4]. The endometrial biopsy classification may be biased, since there is a high degree of subjectivity in the interpretation of histopathological lesions and variability between the grading experience of pathologists/theriogenologists, and heterogenous endometrial sampling site and tissue characteristics. Thus, caution should be taken when interpreting research data based on endometrial biopsy classification. Currently histopathological examination of mare endometrium biopsy is the only available standardized scientific approach, although not perfect. As an alternative, and/or as a complementary diagnostic approach, the development of less invasive and more reliable techniques, such as blood biomarkers would be desirable.

### 8.1. Endometritis and Endometrosis Are Interconnected

Chronic degenerative changes of the endometrium affect fertility in many ways. Lymphatic lacunae and fibrosis can be related to impaired lymph circulation and removal of fluid from the uterus [3,33,34]. Angiopathies decrease blood flow and perfusion of the uterus which in turn can affect endometrial edema, uterine clearance, glandular function, development of the conceptus, and thus overall fertility [3,69,70]. Vascular degeneration is not limited to endometritis but is found also in myometrial vessels and in large arteries and veins between the circular and longitudinal myometrial layers [71]. The degeneration is associated with the number of foals and with the endometrial grade, but not with age. Elastosis of large vessels may indicate compromised myometrial blood flow and subsequently impaired uterine contractility [71]. This can be one explanation for the finding that susceptible mares have deficient contractility after insemination [41]. The presence and severity of endometrosis and angiosis are correlated indicating that they influence each other. Perfusion disorders probably facilitate the progression of endometrosis [68].

Periglandular fibrosis can affect a single gland or multiple glands (nesting) and can be destructive (degeneration and necrosis of glandular epithelial cells) or non-destructive (epithelial cells intact) [68]. In addition, endometrosis can be classified as metabolically active, when stromal cells around the endometrial glands are oval in shape, the cytoplasm is pale and depict ovoid hypochromatic nuclei. In contrast, when the endometrosis is inactive, the periglandular stromal cells are spindle-shaped with elongated hyperchromatic nuclei [68]. Periglandular fibrosis and cystically dilated glands have been associated with fluid collections and ageing of mares [3,8,33,35]. Prolonged post breeding endometritis led to increase in fibrosis [23] showing that inflammation and fibrosis are somehow connected.

### 8.2. Pathogenesis of Endometrosis

**Figure 1:**
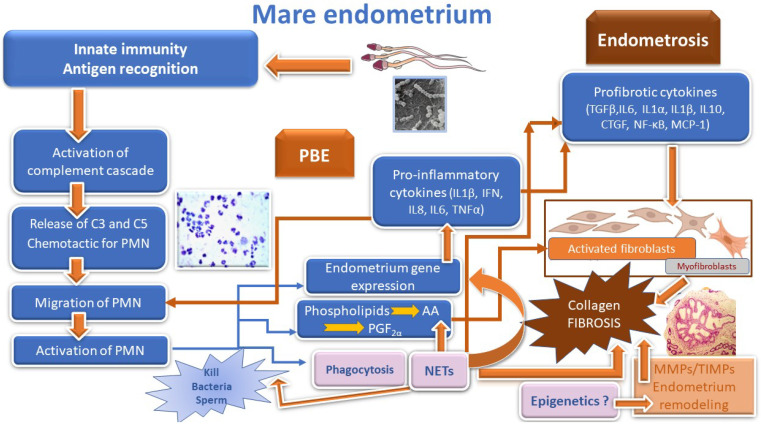
Physiology of post breeding endometritis (PBE) and pathophysiology of endometrosis. C, complement; PMN, polymorphonuclear leukocytes; NETs, neutrophil extracellular traps; AA, arachidonic acid; PGF_2α_, prostaglandin F_2α_; MMPs, matrix metalloproteinases; TIMPs, tissue inhibitors of metalloproteinases; IL, interleukin; IFN, interferon; TNFα, tumor necrosis factor α; TGFβ, transforming growth factor β; CTGF, connective tissue growth factor; NF-κB, nuclear factor kappa B; MCP-1, monocyte chemoattractant protein-1.

Pathogenesis of endometrosis and its association with PBE is depicted in Figure 1. The complex endometrial regeneration during the estrous cycle and pregnancy is similar to the repair processes occurring after tissue damage in other organs or in several pathological conditions [72]. There is a concerted cross-talk among the players throughout the estrous cycle in the mare endometrium, but it is still controversial what drives some mares’ endometrium to develop a pathological condition named endometrosis. The microscopic hallmark of endometrosis is pathological accumulation of collagen (COL) in the lamina propria of the endometrium, mostly as a concentric disposition of stromal cells and/or collagen around the affected endometrial glands, as well as under the basement membrane of the surface epithelium [3,68]. Most recently, the proteomic analysis of uterine lavage fluid of mares with endometrosis has shown that endometrial glandular function is also affected, resulting in the impairment of the secretion of essential proteins [73]. This endometrial dysfunction might hinder early conceptus development, thus contributing to pregnancy loss and infertility [3,74,75].

Even though the relationship between endometrial fibrosis, aging, and infertility has been well established, the etiology of endometrosis being solely ascribed to “wear and tear” and chronic inflammation of the endometrium remains controversial [3,76,77,78]. In fact, it has been reported that aged maiden mares whose endometria had not been challenged with semen, post-breeding endometritis, pregnancy, foaling or post-partum uterine involution developed advanced endometrosis [36].

Mare aging, but not so much parity, is associated with the severity of endometrosis [36,76,79]. It appears that as mares age, dysfunction of modulators of the immune system or of tissue remodeling, such as defensin β, clusterin, uterine serpin, complement C3, neutrophil gelatinase-associated lipocalin (NGAL), or connective tissue growth factor (CTGF), among others, indirectly impair the extracellular matrix (ECM) homeostasis [73,80], which might predispose to fibrogenesis. In addition, mare’s aging has been associated to increased COL deposition in the equine endometrium and in the oviduct [81], and to deficient development of placental microcotyledons [82]. Since equine conceptus development relies initially on the nutrients that derive from exocrine secretions of endometrial glands (histotroph), and later on the placenta, endometrium and placenta’s health are intertwined [83]. A study was carried out in the laboratory of Ferreira-Dias and her group to evaluate COL in placenta from young and older mares [84]. Although COL increased in the pregnant horn of the placenta of mares between 10 and 15 years of age, it did not appear to impair fertility [84]. However, in this study the maximum age of the mares was 15 years, the age after which mares are more likely to develop endometrosis, placental malfunction, and placenta with histopathological lesions related to infertility [36,85]. Aged mares are more prone to develop fibrosis not only in the endometrium but also in the oviduct, which may impair oviductal function, endometrial glandular function, fertilization, early conceptus development, and implantation [3,74,75,86].

#### 8.2.1. Cytokines and Other Fibrosis Mediators

As discussed before, the influx of systemic inflammatory cells, such as neutrophils, into the uterine lumen, and the release of their inflammatory by-products is a major part of the innate immune defense. However, if the PBE turns to PPBE, it may result in the establishment of endometrosis [23]. The inflammatory cells and injured cells in the endometrium produce pro-fibrotic cytokines, chemokines, interleukins, growth factors, and other proteins, which in a paracrine fashion activate resident fibroblasts to differentiate into myofibroblasts leading to fibrogenesis. Besides acting on collagen and other ECM components, endometrial immune cells, such as neutrophils, eosinophils, lymphocytes, resident macrophages, and mast cells synthesize pro-fibrotic cytokines that seem to affect metalloproteinases (MMPs) and their tissue inhibitors (TIMP) in mare endometrium [55,87,88]. These cell signaling paths have been shown to connect inflammation to fibrosis in human kidney [89]. The severity of endometrosis may depend on the effect of transforming growth factor β1 (TGF)-β1, since myofibroblast differentiation is stimulated in vitro by this protein, which is secreted mainly by endometrial immune cells [87,88]. In vitro treatment of equine endometrial fibroblasts with TGF-β1 up-regulated the expression of ECM components and alpha smooth muscle actin (αSMA) transcripts via its effect on MMPs and TIMPs [87,89]. Furthermore, the expression of IL6, IL1α,, IL1β, and IL10 has been linked to inflammatory cells (lymphocytes, neutrophils, eosinophils), and to histopathological lesions of the endometrium, being up-regulated in the presence of endometrosis [87,90,91]. In addition, in the mare, TGF-β1 might be an important regulator of the remodeling of the endometrial ECM mediated by MMPs and TIMPs [89].

A small phospholipid molecule, named lysophosphatidic acid (LPA), modulates cellular interactions, which are crucial for many physiological processes and cytoskeleton structure maintenance [92]. Also in the mare, LPA was found in the endometrium in all phases of the estrous cycle, but in higher levels in the mid-luteal phase. In the follicular phase, LPA content was lower in the presence of endometrosis, when compared to healthy endometrium [93]. In addition, when mid-luteal phase endometrial explants were treated with LPA, there was an increase in the in vitro production of CTGF from Kenney and Doig’s category IIB and III explants [4,93]. As shown in a mouse peritoneal fibrosis model, LPA contributes to fibrosis by stimulating CTGF and driving fibroblast proliferation in a paracrine manner [94]. Therefore, LPA and CTGF could affect physiological events of the endometrium throughout the estrous cycle and early pregnancy [93]. Another hormone-dependent activation of the NF-κB-dependent fibrosis pathway has been reported to occur in the follicular phase, under estrogen dominance [95]. The canonical NF-κB signaling pathway was activated in the follicular phase when an active destructive endometrosis was present [96]. Nevertheless, when destructive endometrosis was inactive, activation of this fibrotic pathway only occurred in the mid-luteal phase [96]. In addition, pro-inflammatory monocyte chemoattractant protein-1 (MCP-1) gene transcripts were up-regulated in destructive endometrosis [96]. Thus, several cytokines and growth factors could affect the remodeling of the ECM, and immunomodulate fibrogenesis in the establishment of endometrosis in the mare [55].

#### 8.2.2. Neutrophils and NETs

Neutrophils, as the first line of the non-specific innate immune defense mechanism against pathogens, move from the bloodstream, strongly attach to the endothelium cell barrier to cross it and to get to the infection site in the tissue. There neutrophils are activated by chemokines produced by mast cells and resident tissue macrophages to kill infectious agents in many diverse ways [97,98]. In addition to their classical functions of phagocytosis and degranulation by the extracellular release of lytic enzymes and the capacity to form reactive oxygen species (ROS) by NADPH oxidase, these inflammatory cells have developed a phagocytosis-independent system of pathogen destruction by forming neutrophil extracellular traps—NETs [52,99,100]. The NETs consist of the extracellular release of DNA strands surrounded by proteins from neutrophil cytoplasm and nucleus, which entangle bacteria and parasites to destroy them [52,97]. Also, the mare endometrium, when challenged by bacteria, such as strains of *Escherichia coli*, *Streptococcus equi* subspecies *zooepidemicus* or *Staphylococcus capitis* isolated from mares with endometritis, has the ability to form NETs, both in vitro and in vivo, as shown by their presence in endometrial mucus ex vivo [101]. The proteins found in NETS, such as histones, myeloperoxidase, cathepsin G, and elastase [52], appear to be an additional antimicrobial mechanism developed in mares to resist endometritis [53,101,102]. Nevertheless, despite the role of neutrophils by restraining the infectious agents in situ through NETs action, their persistence might result in tissue damage and COL deposition and fibrosis establishment [53,102]. Likewise, in mare endometrium, explants subjected in vitro to different doses of elastase, myeloperoxidase, or cathepsin G increased COL1 production [103]. All enzymes present in NETs raised in vitro COL1 production by endometrial explants in the follicular phase, and in all Kenney’s endometrium categories, but in mid-luteal phase, only endometria with moderate or severe lesions responded to elastase and cathepsin G with increased COL1 production [103]. Equine neutrophils have also the capacity to produce NETs when in contact with equine spermatozoa, which can be degraded by DNA present in the seminal plasma [104,105]. In contrast, in the donkey, seminal plasma but not sperm cells themselves, induces NETs production [106]. Thus, NETs action could represent a beneficial reproductive strategy for the female reproductive tract to select sperm [106] or fight endometritis [102]. Nevertheless, the persistence of activated neutrophils in mare’s endometrium might have a deleterious effect mediated by NETs enzymes that stimulate fibrosis establishment during estrous cycle and depend on histopathological condition [103].

#### 8.2.3. Prostaglandins

Prostaglandins participate in a variety of physiological functions in female reproduction and are profusely produced in the ovary and endometrium [107]. The role of PGs, their pathway enzymes and tissue receptors in fibrogenesis in human tissues have been reviewed. While PGE_2_ is considered an anti-fibrotic mediator that acts through its receptors 2 (EP2) and 4 (EP4) [108,109], PGF_2α_ mediates COL deposition [110]. It appears that in mare endometrium, PGE_2_ protects against fibrosis induced by NETs enzymes, through its receptor EP2, but not EP4 [111]. Most likely, when anti-fibrotic effect of PGE_2_ is suppressed in the endometrium, due to impaired EP2 expression or PGE_2_ production, fibrogenesis may override. Then, the pathological deposition of COL increases in moderate to severe endometrosis, in the follicular phase, as well as in healthy endometrium or with slight inflammation in mid luteal phase [111]. Also, profibrotic cytokines may be involved in alternative pathways of fibrogenesis, rather than PGs [111].

In contrast, PGF_2α_ acting on its receptor PTGFR has been considered a fibrogenesis mediator in human lung [112] and rat heart fibroblasts [113]. In the mare, endometrial explants treated with enzymes found in NETs, the production of PGF_2α_ and *PTGFR* transcription differed with estrous cycle phase and endometrial category [114]. Thus, NETs enzymes up-regulated PGF_2α_ production and/or PTGFR transcription in the follicular phase endometrium, while in the mid luteal phase tissues with no pathological changes, or very mild ones, PTGFR transcripts decreased [114]. However, when endometrium retrieved in the mid-luteal phase presented endometrosis, enzymes found in NETs induced PTGFR transcripts [114]. As in other tissues, where PGF_2α_ pathway activation facilitates fibrogenesis, PGF_2α_ may also be involved in endometrosis pathogenesis. In the mare, endogenous endocrine priming, such as estrogens in the follicular phase and/or progesterone in the luteal phase, might regulate the PGF_2α_ pathway and stimulate fibrosis establishment in healthy or pathological endometrium challenged by enzymes present in NETs [114]. Thus, an association between the PGF_2α_-pathway and collagen deposition in mare endometrium is suggested [103,114]. In addition, previous in vitro studies in mare endometrium have already evidenced an association between COL1 and PGF_2α_ [115], namely after stimulation of endometrial explants with elastase [116]. Moreover, the increase in COL1 secretion from fibroblasts challenged with PGF_2α_ shows the pro-fibrotic role of this eicosanoid in endometrosis [93]. In the process of endometrosis formation, disruption in the transcript levels of PG synthases and PG production in the endometrium might contribute to estrous cycle dysregulation and to early embryonic loss [117]. In another study, treatment of endometrial fibroblasts with PGE_2_ up-regulated the transcription of MMP-2 and MMP-9 and down-regulated MMP-13 [93]. Since equine spermatozoa induce PGF_2α_ release from the endometrium, which induces neutrophil chemotaxis into the uterus [48], the mechanism of NETs formation might further stimulate PGF_2α_ release. Therefore, PPBE might be responsible for the establishment of endometrosis, mediated by PGF_2α_.

## 9. Epigenetics

Transcript levels of DNA methylases (DNMTs) and their correlation with COL transcripts in the endometrium of mares with different degrees of fibrosis have been evaluated [118]. In the last stage of endometrosis (category III), DNMT3B and COL3A1 transcripts were positively correlated, suggesting that there is a disturbance in collagen and DNMTs in endometrium during the fibrosis process [118]. As previously reviewed for epigenetics influence on idiopathic pulmonary fibrosis in humans [79], the increase in DNMTs might be related to a downregulation of anti-fibrotic genes, thus stimulating fibrogenesis in mare endometrium. The aging process influences DNA methylation, which might have pathologic consequences, such as fibrosis, when DNA in collagen genes is hypermethylated [119,120]. As mares aged, severity of endometrosis, as well as *DNMT1* and *DNMT3B* transcripts increased, accompanied by alterations in the correlation between collagen types and *DNMTs* [118]. However, since DNA methylation only reflects global methylation, identification of specific methylation sites in mare endometrium is imperative to unravel if that occurred in a particular site of a gene (CpG Islands), such as COL.

## 10. Conclusions

A plethora of evidence has shown that endometritis and endometrosis are linked processes that create a vicious cycle. The more prone the mare is to develop endometritis, the more susceptible she is also for endometrosis establishment. Endometrial tissue insult by sperm or pathogens, activation of either resident inflammatory cells, or invading neutrophils from the bloodstream, stimulate the release of pro-inflammatory cytokines and NETs and activation of some pro-fibrotic pathways, either through PG, cytokines, growth factors or epigenetics, affecting ECM architecture and endometrial function. When endometritis prevails, the uterine milieu changes from an inflammatory environment to a fibrotic endometrium, which, in turn, is more susceptible to develop persistent endometritis and to become hostile to sperm and early embryos, resulting in infertility.

## Figures and Tables

**Table 1:** Classification and treatment of endometrial conditions.

Condition	Duration	Treatment	Justification for Treatment
post-breeding endometritis (PBE)	≤24 h	none	physiological defense mechanism
prolonged post-breeding endometritis (PPBE)	>24 h	ecbolics, lavage, immune modulators	presence of intrauterine fluid and neutrophils
acute infectious endometritis	days	antimicrobials, ecbolics, lavage, mucolytics	bacteria or fungi cultured
chronic infectious endometritis	from one to several weeks	antimicrobials, ecbolics, lavage, mucolytics	bacteria or fungi cultured
endometrosis	years	none	degenerative changes
